# Inhibition of Protein-Tyrosine Phosphatase PTP1B and LMPTP Promotes Palmitate/Oleate-Challenged HepG2 Cell Survival by Reducing Lipoapoptosis, Improving Mitochondrial Dynamics and Mitigating Oxidative and Endoplasmic Reticulum Stress

**DOI:** 10.3390/jcm9051294

**Published:** 2020-05-01

**Authors:** Lynda Bourebaba, Jacek Łyczko, Michalina Alicka, Nabila Bourebaba, Antoni Szumny, Andrzej M. Fal, Krzysztof Marycz

**Affiliations:** 1 Department of Experimental Biology, Faculty of Biology and Animal Science, Wrocław University of Environmental and Life Sciences, Norwida 27B, 50-375 Wrocław, Poland; michalina.alicka@upwr.edu.pl (M.A.); nabila.bourebaba@gmail.com (N.B.); 2 International Institute of Translational Medicine, Jesionowa, 11, Malin, 55-114 Wisznia Mała, Poland; 3 Department of Chemistry, Faculty of Biotechnology and Food Science, Wrocław University od Environmental and Life Sciences, Norwida 25, 50-375 Wrocław, Poland; jacek.lyczko@upwr.edu.pl (J.Ł.); antoni.szumny@upwr.edu.pl (A.S.); 4 Collegium Medicum, Institute of Medical Science, Cardinal Stefan Wyszyński University (UKSW), Wóycickiego 1/3, 01-938 Warsaw, Poland; amfal@wp.pl

**Keywords:** NAFLD, lipotoxicity, palmitate, GC-MS, PTP1B, LMPTP, MSI-1436

## Abstract

Objectives: Non-alcoholic fatty liver disease (NAFLD) is considered a well-known pathology that is determined without using alcohol and has emerged as a growing public health problem. Lipotoxicity is known to promote hepatocyte death, which, in the context of NAFLD, is termed lipoapoptosis. The severity of NAFLD correlates with the degree of hepatocyte lipoapoptosis. Protein–tyrosine phosphatases (PTP) including PTP1B and Low molecular weight PTP (LMPTP), are negative regulators of the insulin signaling pathway and are considered a promising therapeutic target in the treatment of diabetes. In this study, we hypothesized that the inhibition of PTP1B and LMPTP may potentially prevent hepatocyte apoptosis, mitochondrial dysfunction and endoplasmic reticulum (ER) stress onset, following lipotoxicity induced using a free fatty acid (FFA) mixture. Methods: HepG2 cells were cultured in the presence or absence of two PTP inhibitors, namely MSI-1436 and Compound 23, prior to palmitate/oleate overloading. Apoptosis, ER stress, oxidative stress, and mitochondrial dynamics were then evaluated by either MUSE or RT-qPCR analysis. Results: The obtained data demonstrate that the inhibition of PTP1B and LMPTP prevents apoptosis induced by palmitate and oleate in the HepG2 cell line. Moreover, mitochondrial dynamics were positively improved following inhibition of the enzyme, with concomitant oxidative stress reduction and ER stress abrogation. Conclusion: In conclusion, PTP’s inhibitory properties may be a promising therapeutic strategy for the treatment of FFA-induced lipotoxicity in the liver and ultimately in the management of the NAFLD condition.

## 1. Introduction

Nonalcoholic fatty liver disease (NAFLD) currently represents one of the most common liver diseases and affects millions of people worldwide. Recovering the increasing prevalence of both obesity and diabetes-type 2 (T2DM), NAFLD is mainly associated with insulin resistance (IR) and, frequently, underlying a metabolic syndrome (MetS) condition [1]. NAFLD has affected about 25% of the European population, and its increasing prevalence has been shown to correlate with the increase in blood sugar levels in overweight and/or type 2 diabetes affected people, which clearly reveals its strong relationship with MetS [2]. NAFLD refers to macrovesicular hepatosteatosis that occurs when alcohol consumption is clinically insignificant (< 20 g/d) and correlates with the non-existence of a viral infection, toxins, autoimmune diseases or congenital metabolic disorders. However, the condition has been largely linked to IR, malnutrition or the consequences of jejuno–ileal bypass surgery. NAFLD can manifest as a simple steatosis or can progress to steatohepatitis, steato-fibrosis, cirrhosis—or even develop into hepatocellular carcinoma (HCC) [3].

Like virtually all metabolic affections, NAFLD is closely and significantly associated with obesity, which is considered to be a chronic inflammatory condition of adipose tissue that secretes multiple cytokines, hormones and lipids that exhibit broad-based metabolic effects. The dysregulation of lipid metabolism is at the heart of this syndrome. The elevated serum free fatty acid (FAA) levels in the state of IR suggest that this state contributes strongly to the onset of obesity and diabetes through the alteration of glucose and lipid metabolism, as well as the appearance of inflammatory cascades [4]. Visceral adipose tissue thus generates multiple signals that may dysregulate cellular metabolism, leading to hepatic fat accumulation and triggering a proinflammatory milieu that induces cellular injury in the liver and other tissues. Failure to suppress injurious processes, such as oxidative stress, dysregulation of the unfolded protein response (leading to endoplasmic reticulum stress), lipotoxicity and apoptotic pathways, strongly contributes to liver damage and progressive fibrosis, which can lead to cirrhosis and the development of hepatocellular cancer in some patients [5]. MetS, which usually includes increased waist circumference, hyperglycemia, dyslipidemia and systemic hypertension (HTN), is also considered to be a prominent risk factor for NAFLD and nonalcoholic steatohepatitis (NASH). The association between NAFLD and MetS features can be bidirectional, particularly with respect to diabetes and HTN, which means that MetS not only increases the risk of NAFLD but can also improve many MetS features and comorbidities [6].

Many physiological factors have been implicated in the emergence and orchestration of metabolic disorders affecting the homeostasis of liver tissue. Among them, PTP1B, which is encoded by the protein tyrosine phosphatase non-receptor type 1 (PTPN1) gene, is one of the most abundant and ubiquitous prototypes of intracellular non-receptor protein tyrosine phosphatases (PTPs) [7]. It has been established for many years that this enzyme may be a pivotal regulator and valuable therapeutic target in different metabolic conditions, such as obesity and type 2 diabetes, through the promotion of insulin resistance, lipogenesis and endoplasmic reticulum stress [8]. In this direction, many pharmacological PTP1B inhibitors have been proposed and have already demonstrated promising properties for the management of these issues [9]. Many investigations have demonstrated a critical link between PTP1B and the liver-specific features of the MetS. To this end, total PTP1B protein levels were found to be significantly elevated in liver biopsies from patients with non-alcoholic steatohepatitis [10]. Furthermore, the hepatocytes of fructose-fed hamsters, in a model of insulin resistance and fatty liver disease, exhibited significant overactivity and higher concentrations of PTP1B protein compared to normal-fed animals [11]. Additionally, other studies have reported that the disruption of liver PTP1B expression positively potentiates hepatic insulin signaling, significantly improves insulin suppression of hepatic glucose production, decreases serum and hepatic triglyceride and cholesterol levels, and alleviates ER stress in a liver-specific PTP1B−/− mice model fed with a high-fat diet [12,13]. Moreover, the evidence that PTP1B is responsible for the dephosphorylation of Janus Kinase 2 (JAK2), the insulin receptor (INRS) and the insulin receptor substrate (IRS) also suggest that PTP1B may be strongly involved in regulating both the insulin and leptin signaling pathways [14,15]. Low molecular weight protein tyrosine phosphatase (LMPTP), also known as acid phosphatase locus 1, is a cytosolic enzyme belonging to the PTPases family, and encoded by the *Acp1* gene, that is widely expressed in various mammalian tissues, with a predominant localization in liver and brain [16].

Considerable lines of evidence support the prominent contribution of LMPTP in modulating glucose and lipid metabolism during obesity and diabetes, as abnormal LMPTP regulation has been reported in animals and patients exhibiting important metabolic dysfunctions, such as insulin resistance (IR) [17,18,19]. Previous *Acp1* silencing resulted in lowered hyperlipidemia incidence in obese patients, as well as reduced glycemic levels in diabetic individuals [20,21,22]. LMPTP knockdown in diet-induced obese C57BL/6 (B6) mice also enabled the improvement of glycemic profile through IR alleviation, and enhanced INSR phosphorylation in mouse hepatocytes and adipocytes [23]. Moreover, overexpression of catalytically inactive recombinant LMPTP in immortalized mouse fibroblasts engendered a restoration of insulin-induced INSR tyrosine phosphorylation, indicating that LMPTP regulates insulin cascades through its phosphatase activity [24]. Based on these data, it has been postulated that PTP1B and / or LMPTP inhibitors may also be convenient for the treatment of fatty liver disorders, such as NAFLD. A recent study reported on the use of dietary supplements, such as curcumin and other natural compounds like the antioxidant resveratrol, for the effective inhibition of PTPs at both the mRNA and protein levels, resulting in the prevention of hepatic steatosis and the restoration of insulin sensitivity in both fructose-fed rats and hyperglycemic IRS2−/−mice [25,26]. Trodusquemine, also known as MSI-1436, is a natural spermine-cholesterol adduct that was shown to potently inhibit PTP1B via a novel mechanism. MSI-1436 acts as a specific, reversible and non-competitive inhibitor of PTP1B through preferential targeting of the long form of PTP1B(1–405), which contains an extended C-terminal segment. Moreover, MSI-1436 showed its ability to attenuate PTP1B-induced HER2-dependent tumorigenesis in vivo [27].

For its part, selective LMPTP inhibition has been achieved using the N,N-diethyl-4-(4-((3-(piperidin-1-yl)propyl)amino)quinolin-2-yl) benzamide or Compound 23, which demonstrated potent abilities in reversing high-fat diet-induced diabetes in mice, through a direct action on the liver, recapitulating the phenotype of mice carrying global or liver-specific LMPTP deficiency [28]. 

The aim of this study was to investigate whether inhibiting liver-PTP1B and LMPTP in human hepatocytes with MSI-1436 and compound 23 (N,N-diethyl-4-(4-((3-(piperidin-1-yl)propyl)amino)quinolin-2-yl) benzamide), over the course of an experimental lipotoxic status induced by a combination of two free fatty acids (namely, palmitate and oleate) can protect cells from lipoapoptosis, oxidative stress, mitochondrial dysfunction and endoplasmic reticulum stress, which are salient features of NAFLD.

## 2. Materials and Methods

### 2.1. Cell and Culture Conditions

The human hepatocarcinoma HepG2 cell line (ATCC® HB-8065™) was purchased from the American Type Culture Collection (Manassas, VA, USA) and was cultured in low-glucose Dulbecco’s modified Eagle’s medium (DMEM, Gibco Carlsbad, CA, USA) supplemented with 10% (v/v) heat inactivated fetal bovine serum (FBS, Gibco Carlsbad, CA, USA) and 2 mM glutamine (Gibco Carlsbad, CA, USA). The cultures were maintained at 37 °C in a 95% humidified 5% CO_2_ atmosphere. Cells were subcultured when they reached 70–80% confluence every 3 days using a Trypsin/EDTA solution (Gibco Carlsbad, CA, USA).

### 2.2. FFA/BSA Complex Preparation and Related Cell Treatments

In order to evaluate the beneficial outcomes of PTP1B and LMPTP inhibition on FFA-induced lipotoxicity, the HepG2 cells used in the present experiments were preconditioned with two well established PTP1B and LMPTP inhibitors, Trodusquemine (MSI-1436, Sigma Aldrich Poznań, Poland) and N,N-diethyl-4-(4-((3-(piperidin-1-yl)propyl)amino)quinolin-2-yl)benzamide (Compound 23, University of California San Diego, La Jolla, CA, USA), both at 1 µM for 24 h under standard culture conditions. The control groups were maintained under the same conditions without inhibitor supplementation.

To induce FFA overloading, treated and untreated HepG2 cells were subsequently exposed to a mixture of long-chain FFAs (oleic acid/palmitic acid, 2:1, Sigma Aldrich, Poznań, Poland) at a final concentration of 2 mM for an additional 24 h. Both fatty acids were complexed with bovine serum albumin (BSA, Sigma Aldrich, Poznań, Poland) at a molar ratio of 2:1 prior to their addition to an FBS-free culture medium. A BSA supplemented medium was used for all control conditions. The selected fatty acid concentration reflects the pathological affection induced by circulating fatty acids (from both free and triglyceride-rich lipoprotein derived sources) observed over the course of obesity, metabolic syndrome and NAFLD [29].

### 2.3. Cytotoxicity Assay

To determine cell viability, HepG2 cells were seeded at known densities and incubated under control or high fatty acid conditions for 24 h following pretreatment with both inhibitors for 24 h, as indicated. The cell growth rate was then evaluated using a 10% resazurin-based dye-TOX-8 (Sigma Aldrich, Poznań, Poland) solution according to the manufacturer’s protocols. In brief, the media were collected and replaced with the culture medium containing 10% resazurin dye solution. Then, the cultures were incubated with dye in a CO_2_ incubator at 37 °C for 2 hours. Thereafter, absorbance was measured at a wavelength of 600 nm for resazurin and at the 690 nm reference wavelength using a microplate reader (BMG Labtech, Germany). Each test included a blank (dye containing medium without cells) as a control.

### 2.4. Oil Red O Staining

The intracellular accumulation of neutral lipids within the treated and untreated HepG2 cells was evaluated by Oil Red O staining. Briefly, the cells were fixed in 4% paraformaldehyde for 45 min at room temperature followed by incubation with 60% isopropanol for 5 min. The fixed cells were afterwards stained with 0.5 g/mL Oil Red O (Sigma Aldrich, Poznań, Poland) in 60% aqueous isopropanol for 15 min at room temperature and then rinsed with 60% aqueous isopropanol and PBS. The nuclei were subsequently counterstained with hematoxylin (Sigma Aldrich, Poznań, Poland) for 1 min. The cells were observed under an inverted microscope (AxioObserverA1, Zeiss, Oberkochen, Germany), and pictures were acquired using a Canon PowerShot digital camera (Canon, Woodhatch, UK).

### 2.5. Fluorescent Detection of Intracellular Lipid Droplets

The accumulation of neutral lipid droplets was analyzed using an HCS LipidTOX™ Green Neutral Lipid Stain (Invitrogen Life Technologies, Warsaw, Poland) for cellular imaging. Staining was performed in accordance with the manufacturer’s instructions. Briefly, all treated and untreated HepG2 cells were fixed with 4% paraformaldehyde for 40 min at room temperature, washed three times with HBSS (Sigma Aldrich, Poznań, Poland), and labeled with LipidTOX™ Green for 20 min at room temperature. Subsequently, the nuclei were counterstained with 4′,6-diamidino-2-phenylindole (DAPI) using the ProLong™ Diamond Antifade Mountant with DAPI (Invitrogen Life Technologies, Warsaw, Poland). Photomicrographs were captured using a confocal microscope (Observer Z1 Confocal Spinning Disc V.2 Zeiss with a live imaging chamber). The obtained photomicrographs were then merged and analyzed using the ImageJ software (Bethesda, MD, USA).

### 2.6. Flow Cytometric Analysis of Early Apoptosis

In cultured human HepG2 hepatoma cells, the early stages of apoptosis were scored by detecting the exposure of phosphatidylserine (PS) on the cell membrane. An Annexin-V and Dead Cell Assay kit™ (Cat. No. MCH100105, Merck Millipore, Darmstadt, Germany) was used to reveal the PS exposure via a Muse Cell Analyzer (Merck Millipore, Darmstadt, Germany). Following the supplier’s instructions, all treated and untreated groups of cells were collected by trypsinization, washed with HBSS and labeled with an Annexin V & Dead Cell Kit for 20 min at room temperature. The apoptotic ratio was calculated by identifying four populations: (i) non-apoptotic cells for those not undergoing detectable apoptosis: Annexin V (−) and 7-AAD (−); (ii) early apoptotic cells, Annexin V (+) and 7-AAD (−); (iii) late apoptotic cells, Annexin V (+) and 7-AAD (+); and (iv) cells that have died through a non-apoptotic pathway: Annexin V (−) and 7-AAD (+).

### 2.7. Quantification of Multicaspase Activity

Multicaspase activity was quantified using a Muse MultiCaspase assay kit (Cat. No. MCH100109, Merck Millipore, Darmstadt, Germany). HepG2 cells were treated with MSI-1436 and Compound 23 inhibitors prior to the palmitate/oleate overloading process. Multicaspase activity was subsequently assessed according to the manufacturer’s instructions using a Muse Cell Analyzer (Merck Millipore, Darmstadt, Germany).

### 2.8. Mitochondrial Transmembrane Potential

The mitochondrial transmembrane potential (ΔΨ) was measured using a cationic and lipophilic fluorescent probe, which accumulates within high potential membranes under high fluorescence and decreases proportionally with depolarization of the mitochondrial membranes. HepG2 cells were treated with both tested PTP inhibitors for 24 h and then exposed to 2 mM of FFAs for another 24 h. The cells were then collected, washed with HBSS and stained with a Muse™ MitoPotential Assay kit (Cat. No. MCH100110, Merck Millipore, Darmstadt, Germany) as recommended by the manufacturer. The percentage of total depolarized cells (depolarized live + depolarized dead cells) was established by the mean of a Muse Cell Analyzer (Merck Millipore, Darmstadt, Germany).

### 2.9. Measurement of Intracellular ROS

The percentage of cells undergoing oxidative stress as defined by the overproduction of ROS (namely, superoxides) was established by a Muse® Oxidative Stress Kit (Cat. No. MCH100111, Merck Millipore, Darmstadt, Germany). Briefly, after culturing and treatment, HepG2 cells were washed with HBSS and suspended in a 1X assay buffer (Muse® Oxidative Stress Kit, Merck Millipore, Darmstadt, Germany). Thereafter, all samples were incubated for 30 min at 37 °C and then the ROS positive cells were monitored using the Muse® Cell Analyzer Merck Millipore, Darmstadt, Germany.

### 2.10. Protein Carbonylation Assay

The concentration of protein carbonyl moieties was determined using the DNPH (2,4-dinitrophenyl hydrazine) derivatization method. HepG2 cells were seeded onto 6-well plates and treated with two PTP inhibitors for 24 h. After FFA mixture exposure, the cells were harvested, washed with HBSS, and the total protein level was measured from the cell lysates using a Bicinchoninic Acid Kit ^®^ (Sigma Aldrich, Poznań, Poland). Quantification of the carbonyls was performed using a Protein Carbonyl Content Assay Kit (Cat. No. MBS841906, MyBioSource, San Diego, CA, USA) following the manufacturer’s instructions. The results were expressed as the nmol of carbonyl / mg of protein.

### 2.11. Gene Expression Analysis

The gene expression levels of the main transcripts involved in apoptosis, ER stress and mitochondrial dynamics (Table 1) were assessed by means of Real-Time Quantitative Reverse Transcription PCR. Total RNA was isolated from HepG2 cells at the end of each experiment using a Trizol reagent (Sigma, St. Louis, MO, USA) as recommended by the supplier. RNA purity and concentration were measured using a nanospectrophotometer (WPA, Biowave II, Germany). Genomic DNA (gDNA) digestion and cDNA synthesis were performed via a reverse transcription reaction with oligo (dT) primers using a Tetro cDNA Strand cDNA Synthesis Kit (Bioline, London, UK) in a T100 Thermal Cycler (Bio-Rad, Hercules, CA, USA) according to the provided kit instructions. A SensiFAST SYBR Green Kit (Bioline, London, UK) was used to detect the target mRNA expressions in a CFX Connect™ Real-Time PCR Detection System (Bio-Rad). A total of 150 ng of cDNA was amplified in a total volume of 10 µl containing a SYBR-Green Master Mix, forward and reverse primers and tested samples. The thermal profile conditions were as follows: 95 °C for 2 min followed by 40 cycles at 95 °C for 15 seconds, annealing for 15 seconds and elongation at 72 °C for 15 seconds. RT-qPCR reactions were carried out in triplicate. The relative expression levels of all tested genes were normalized to the expression of the house-keeping gene glyceraldehyde-3-phosphate dehydrogenase (GAPDH). Expression of the splicing of XBP1 was estimated by standard quality RT-PCR using agarose electrophoresis. The PCR products were run in 2% gel and separated to visualize a 26 bp shift.

### 2.12. Metabolomic Profiling of HepG2 FAAs

#### 2.12.1. Cell lysis and Metabolite Extraction

The HepG2 FAAs from all treated and untreated groups were isolated using a mono-phasic mixture of chloroform/methanol/water according to the procedure described by Bai and collaborators [30]. Briefly, 1 ml of chloroform/methanol/water mixture in a ratio of 20:50:20 was added to each sample in a chemical fume hood. Next, cells were subjected to ultra-sonication at room temperature with extraction reagents in a water bath sonicator for 100 min and vortexed for 2 min. Thereafter, the samples were transferred to 1.5 ml centrifugation tubes and centrifuged at 4 °C, 18,000×g for 20 min. Afterwards, the supernatants were collected for each sample and dried completely overnight.

#### 2.12.2. Preparation of HepG2 Fatty Acid Methyl Esters (FAMEs)

The previously obtained HepG2 crude lipid extract was dissolved in 5 ml of 1 M potassium hydroxide in methanol (KOH/MeOH) solution and boiled with reflux for 15 min. Then, after cooling, 5 ml of distilled water was added, and the solution was extracted three times with 2 ml of hexane. The solution and hexane were then collected together and dried over anhydrous magnesium(II) sulfate(VI) (MgSO_4_). Then, the solvent was removed on a rotary evaporator and 5 ml of 14% solution of boron trifluoride in methanol (BF_3_/MeOH) (Sigma-Aldrich, Steinheim, Germany) was added for the methylation process for 15 min at 80 °C. After cooling, 2 mL of distilled water was added and the solution was extracted three times with 2 mL of hexane, which were again combined together. The hexane was washed with saturated NaCl solution and dried over anhydrous MgSO_4_. In the last step, HepG2 FAMEs solution was densified to an approximate volume of 200 μL under nitrogen and transferred to a GC-MS vial with a glass insert.

After the GC-MS analysis, when the absence of the heptadecanoic acid methyl ester (Me. C17:0) (Sigma-Aldrich, Steinheim, Germany) was confirmed, the procedure was repeated with the addition of 20 μg of Me (C17:0 before dissolving the sample in KOH/MeOH solution, as per the internal standard (IS)). The preparations were made in triplicate.

#### 2.12.3. FAME Qualitative and Quantitative Analysis

For the qualitative and quantitative analysis of the HepG2 FAME profile, a Shimadzu GCMS-QP2020 (Kyoto, Japan) equipped with a ZB-FAME (Phenomenex, Torrance, CA, USA) column (60 m × 0.25 mm i.d. × 0.25 µm film thickness) was used. The GC oven temperature was programmed at 80 °C, increased 2 min at 180 °C at a rate of 3.0 °C, and then increased to 240 °C at a rate of 8.0 °C and kept for 4 min. The injection temperature was 280 °C, and helium gas was used as the carrier gas at a flow rate of 1.8 mL·min^−1^. The samples were injected with a split ratio of 1:10. Scanning was performed from 40 to 400 m/z in the electronic impact (EI) mode at 70 eV, while the ion source temperature was 220 °C.

FAMEs were identified via comparison to an analytical standard Supelco 37 component FAME Mix (Bellefonte, PA, USA) analysis carried out under the same conditions, and the experimentally obtained mass spectra were compared to those available in the NIST17 database. The quantification was performed by integrating the peak areas and calculations based on the amount of IS added to the analysis.

### 2.13. Statistics

All statistical analyses were performed using GraphPad Prism (San Diego, CA, USA) with a one-way analysis of variance (ANOVA) followed by Dunnett’s post hoc multiple comparison test, as indicated. Asterisk (*) and Hash (#) signs indicate statistical significance in the FFA-induced groups versus the healthy control or in the FFA-induced control versus the PTP inhibitor-treated groups, respectively. All *p* values lower than 0.05 (*p* < 0.05) are summarized with one asterisk/hash (*/#), those at *p* < 0.01 use two asterisks/hashes (**/##) and those at *p* < 0.001 have three asterisks/hashes (***/###).

## 3. Results

### 3.1. Effects of FFA and PTP Inhibitor Treatments on HepG2 Cell Viability

**Figure 1:**
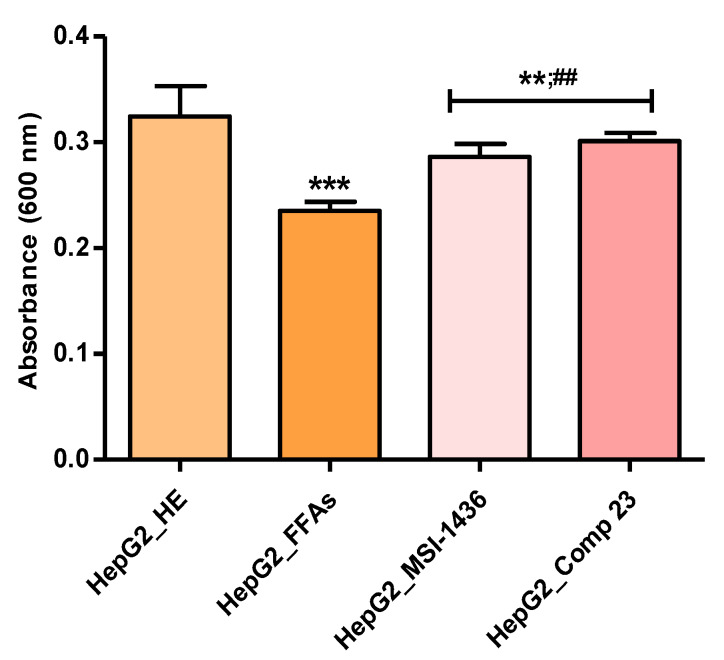
Cytotoxicity in HepG2 cells upon exposure to the palmitate/oleate combination and protein–tyrosine phosphatases (PTP) inhibitor pre-conditioning. Histograms represent the average absorbance of the metabolized resazurin dye at 600 nm after cell exposure. The results are expressed as the mean of 3 different experiments ± SD. Asterisks (*) refer to a comparison of all treated groups to untreated healthy cells. A hashtag (#) refers to a comparison between PTP inhibitor treated groups and FFA-challenged cells. **/##, *p* < 0.01, ***, *p* < 0.001. HepG2_HE: HepG2 healthy untreated cells; HepG2_FFAs: HepG2 cells exposed to the palmitate/oleate combination; HepG2_MSI-1436: HepG2 cells pre-treated with 1 µM MSI-1436 inhibitor and exposed to the palmitate/oleate combination; HepG2_Comp 23: HepG2 cells pretreated with 1 µM compound 23 inhibitor and exposed to the palmitate/oleate combination.

To determine whether the palmitate/oleate combination exerts growth-inhibitory effects in HepG2 cells and to elucidate the possible protective effects of MSI-1436 and Compound 23 on FFA-induced cytotoxicity, the cells were exposed to 2 mM of FFAs for 24 hours, and two groups were pretreated with 1 µM of each PTP inhibitor. Cell growth was evaluated by Resazurin-based TOX8 assays. As shown in Figure 1, the incubation of HepG2 cells with the FFA mixture significantly reduced the average of the metabolically active living cells compared to the healthy untreated HepG2 cells (*p* < 0.001). Twenty-two-hour pretreatment of the HepG2 cells with MSI-1436 and Compound 23 noticeably prevented palmitate/oleate-induced cell growth arrest and cytotoxicity with respect to the FFA-challenged group of cells (*p* < 0.01).

### 3.2. Analysis of Lipid Accumulation in the FFA-Overloaded and Treated Cells

**Figure 2:**
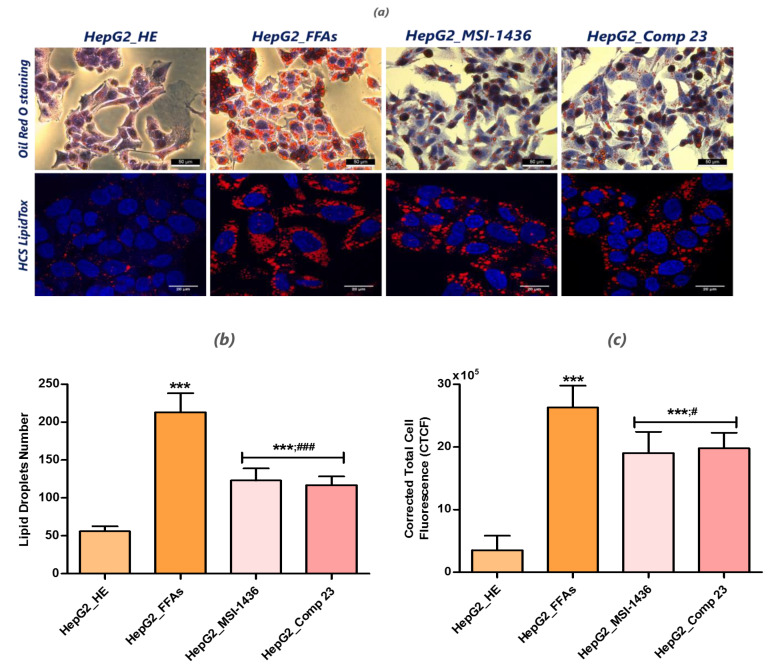
Evaluation of lipid accumulation in HepG2 cells. (**a**) Representative photomicrographs of HepG2 cells treated with FFAs and PTP inhibitors stained with Oil Red O staining at 40-fold magnification and the confocal images of HCS LipidTox stained treated and untreated HepG2 cells; scale bar size 20 µm; magnification was set to 60-fold. (**b**) The histogram summarizes the mean percentage ± SD of three independent lipid droplet quantifications using the ImageJ software. (**c**) A bar chart representation of the corrected total cell fluorescence (CTCF) for HCS LipidTox calculated using the ImageJ software. An asterisk (*) indicates a comparison of all treated groups to untreated healthy cells. A hashtag (#) refers to a comparison of the PTP inhibitor treated groups to the FFA-challenged cells. #, *p* < 0.05, ***/###, *p* < 0.001. HepG2_HE: HepG2 healthy untreated cells; HepG2_FFAs: HepG2 cells exposed to the palmitate/oleate combination; HepG2_MSI-1436: HepG2 cells pre-treated with 1 µM MSI-1436 inhibitor and exposed to the palmitate/oleate combination; HepG2_Comp 23: HepG2 cells pre-treated with 1 µM compound 23 inhibitor and exposed to the palmitate/oleate combination.

After treating the cells with the FFA mixture for 24 hours, the intracellular accumulation of neutral lipids in the HepG2 cells was assessed through Oil Red O and HCS LipidTox staining (Figure 2). The obtained photomicrographs indicate that the FFAs induced significant cellular lipid accumulation *(p <* 0.001) compared to the healthy cells (Figure 2a), which exhibited a lower number of lipid droplets and HCS LipidTox fluorescence intensity (Figure 2b,c). Lipid droplet quantification between the two groups of cells treated with MSI-1436 and Compound 23 showed reduced lipid accumulation with respect to the FFA-treated HepG2 cells *(p* < 0.001) (Figure 2b). Confocal image processing confirmed the preventive effects of the two PTP inhibitors on the intracellular accumulation of palmitate and oleate (Figure 2c), albeit to a lesser extent *(p* < 0.05).

### 3.3. MSI-1436 and Compound 23 Inhibit FFA-Induced Lipo-Apoptosis in HepG2 Cells

**Figure 3:**
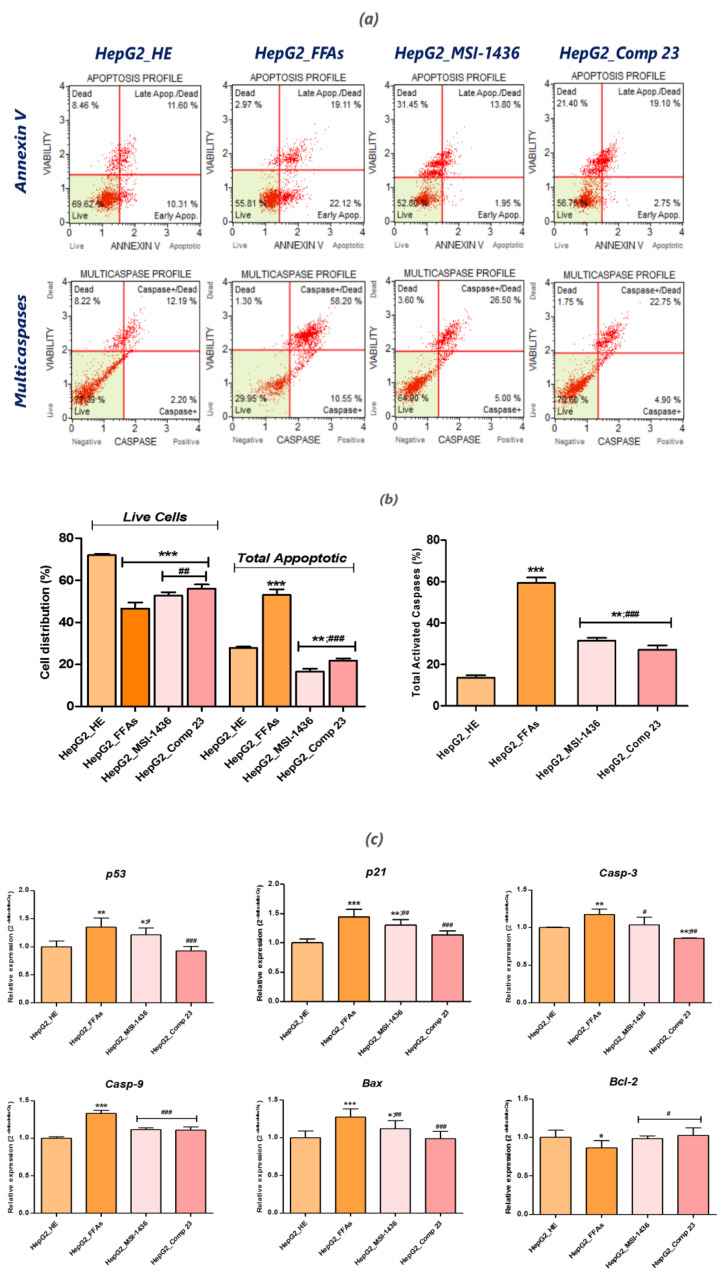
Evaluation of lipoapoptosis in MIS-1436 and compound 23-preconditionned or non-treated HepG2 cells following intracellular free fatty acid (FFA) accumulation. (**a**) Apoptosis and Multicaspase profile plots. Each plot is a representative figure of the three replicates of each determination. (**b**) Bar charts depicting the percentage of live and total apoptotic cells, as well as the average of the total positively activated multicaspase cells. (**c**) Representative bar charts of the relative expression of apoptotic key markers. Representative data from three independent experiments are shown as mean ± SD (*n* = 3). An asterisk (*) indicates a comparison of all treated groups to untreated healthy cells. A hashtag (#) refers to a comparison of the PTP inhibitor treated groups to FFA-challenged cells. */# *p* < 0.05, **/## *p* < 0.01, ***/### *p* < 0.001. HepG2_HE: HepG2 healthy untreated cells; HepG2_FFAs: HepG2 cells exposed to the palmitate/oleate combination; HepG2_MSI-1436: HepG2 cells pre-treated with 1 µM MSI-1436 inhibitor and exposed to the palmitate/oleate combination; HepG2_Comp 23: HepG2 cells pre-treated with 1 µM compound 23 inhibitor and exposed to the palmitate/oleate combination.

To investigate whether cells undergo apoptosis, untreated or FFA-treated cell lines, as well as MSI-1436 and compound 23-preconditionned cells, were evaluated using Muse™ Annexin V and Dead Cell and Muse MultiCaspase™ assay kits for total apoptotic cell determination and caspase activation testing, respectively and using RT-qRCP to establish the main apoptotic-related gene expression profile (Figure 3). The group of cells treated with the FFA complex consisting of palmitate and oleate exhibited a two-fold higher percentage (Figure 3b) of total apoptotic cells (including early and late apoptotic cells) compared to the healthy non-exposed cells (*p* < 0.001). The total caspase activity measurement also highlighted a sharp activation of caspase complexes following FFA supplementation in HepG2 cultures (above 40% relative to the untreated control (*p <* 0.001)). FFA-induced apoptosis was also confirmed through a Rt-qPCR analysis (Figure 3c). The obtained data clearly demonstrate that induction of the main apoptosis related genes takes place in parallel with the onset of apoptosis mediated by the FFAs in HepG2 cells (Figure 3c). All *p53*, *p21*, *Bax*, *Casp-3* and *Casp-9* transcripts were significantly over-expressed following cell FFA-overloading. A relative downregulation of *Bcl-2* expression was also recorded in the same group (*p* < 0.05). When cells were pretreated with both MSI-1436 and compound 23, which are known potent PTP inhibitors, total apoptotic cell rate dropped by over 30% for both inhibitors (Figure 3.b) compared to the FFA-challenged cells *(p <* 0.001). Likewise, treatment of the cells with PTP inhibitors prevented the over activation of the caspase complexes, as evidenced by the average two-fold reduction in caspase activity, contrary to the palmitate/oleate-induced lipoapoptosis in HepG2 cells (*p* < 0.001). Activation of the apoptosis effectors appeared, nevertheless, to be higher than that recorded for the physiologically healthy cells exhibiting normal FFA concentrations (*p* < 0.01). The assessment of the regulatory effects of the tested PTP inhibitors relative to the expression of key apoptosis-related factors confirmed the efficacy of these compounds in abrogating the apoptosis mediated by FFA excess. Indeed, after HepG2 cell preconditioning, the mRNA abundance of pro-apoptotic *p53*, *p21*, *Bax*, *Casp-3* and *Casp-9* genes was markedly down-regulated to basal expression levels compared to both the FFA-treated and normal HepG2 control groups (Figure 3c). Additionally, Compound 23 exhibited the strongest anti-apoptotic effect and was the most effective in regulating the expression patterns of the studied factors. Interestingly, the two PTP inhibitors positively regulated and restored the basal expression of the anti-apoptotic *Bcl-2* gene, thereby promoting HepG2 cell survival in the presence of high intracellular FFA amounts (*p* < 0.05).

### 3.4. MSI-1436 and Compound 23 Improve Mitochondrial Dynamics in HepG2 FFA-Treated Cells

**Figure 4:**
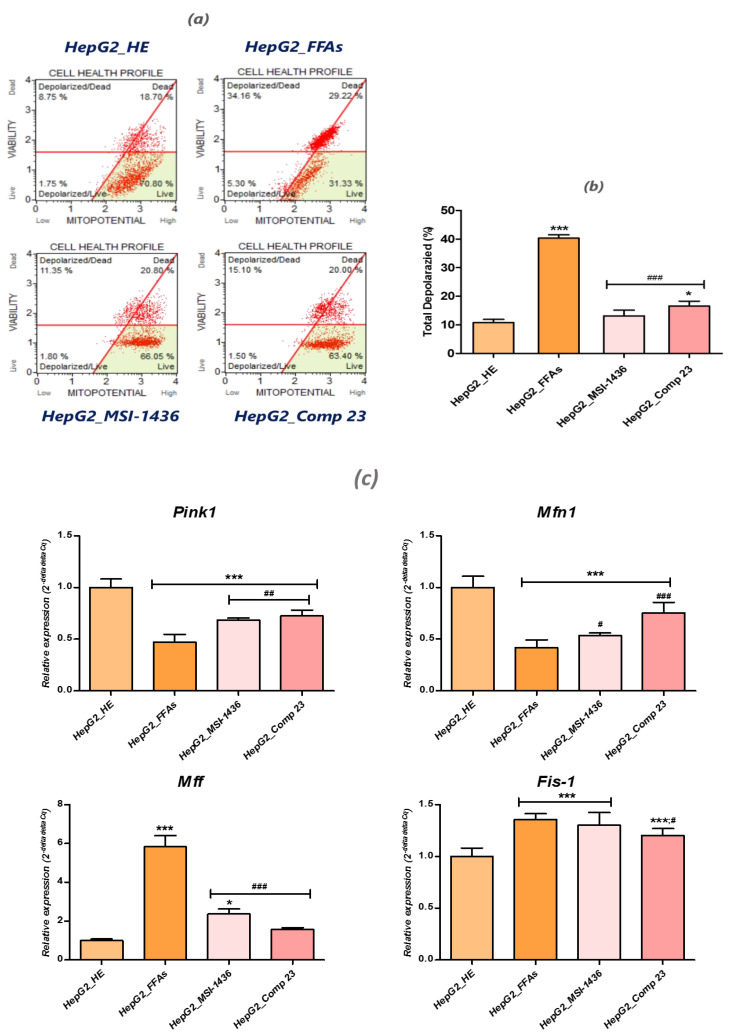
Changes in the mitochondrial dynamic assessment in FFA-challenged HepG2 cells in the presence or absence of MSI-1436 and compound 23 PTP inhibitors. (**a**) A scattered blot representation of the cellular mitochondrial health profile for one representative experiment. (**b**) Bar-charts represent the average percentages ± SD for the total depolarized cells of three repetitions. (**c**) Histograms summarize the relative expression of mitochondrial fusion and fission regulators. Representative data from three independent experiments are shown as mean ± SD (*n* = 3). An asterisk (*) refers to a comparison of all treated groups to untreated healthy cells. A hashtag (#) indicates a comparison of the PTP inhibitor treated groups to FFA-challenged cells. */#, *p* < 0.05, ***/###, *p* < 0.001. HepG2_HE: HepG2 healthy untreated cells; HepG2_FFAs: HepG2 cells exposed to the palmitate/oleate combination; HepG2_MSI-1436: HepG2 cells pre-treated with 1 µM MSI-1436 inhibitor and exposed to the palmitate/oleate combination; HepG2_Comp 23: HepG2 cells pre-treated with 1 µM compound 23 inhibitor and exposed to the palmitate/oleate combination.

To examine whether inhibition of the PTP enzyme accounts for the prevention of mitochondrial disruption and, consequently, the anti-apoptotic effect exhibited by PTP inhibitors, changes in mitochondrial permeability were evaluated using a Muse™ MitoPotential Assay kit. When HepG2 cells were overloaded with the palmitate/oleate combination, the △Ψm was significantly depolarized (*p* < 0.001), as shown by the decrease in the accumulation of MitoPotential dye within the inner membrane of the intact mitochondria (Figure 4b). Pretreatment with MSI-1436 and compound 23 notably reduced the changes in △Ψm and stabilized the mitochondrial transmembrane permeability transition, as indicated by the restoration of the MitoPotential dye fluorescence (Figure 4a). Meantime, alterations in mitochondrial dynamics and abnormal phenotypes involving either excessively fragmented mitochondria or hyper-fused elongated tubule mitigation were studied via a Rt-qPCR analysis of the genes that regulate mitochondrial fission and fusion (Figure 4c). FFA-overloaded cells exhibited lower levels of the fusion mediators *Mfn1* and *Pink1* and markedly higher levels of the mitochondrial fission mediators *Fis1* and *Mff*, than normal healthy HepG2 cells (*p* < 0.001). This finding suggests that the observed mitochondrial dysfunction in lipotoxic HepG2 cells was related to both impaired fusion and enhanced fission (Figure 4c). The pharmacologic enhancement of mitochondrial networking using both MSI-1436 and compound 23 led to remarkable restoration of *Mfn1* and *Pink1* fusion gene expression while down-regulating the tested mitochondrial fission mediators *Fis-1* and *Mff*, where compound 23 exerted more effective activity than MSI-1436 (Figure 4c).

### 3.5. MSI-1436 and Compound 23 Reduce the Oxidative Stress Generated in HepG2 FFA-Treated Cells

**Figure 5:**
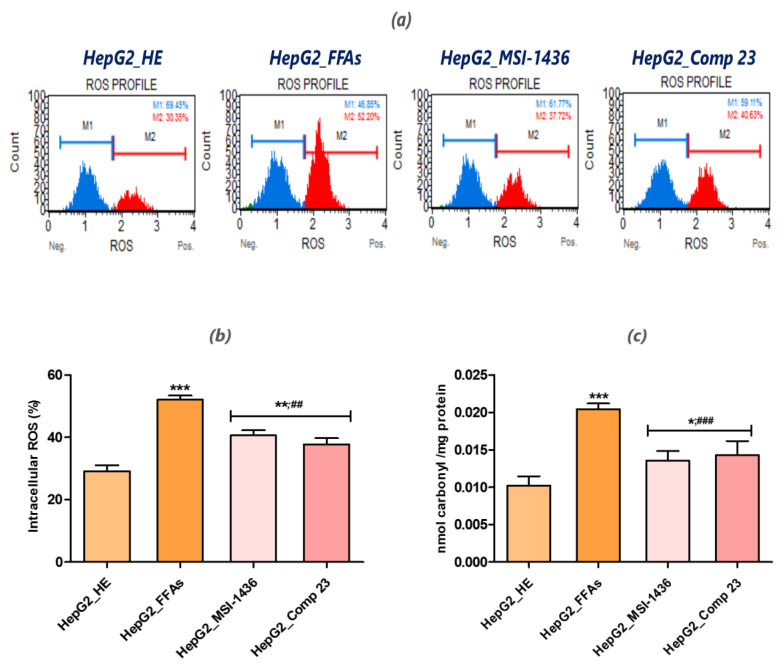
Effects of MSI-1436 and compound 23 on the palmitate/oleate-induced reactive oxygen species (ROS) production and protein carbonylation in HepG2 cells. (**a**) Representative plots depicting cells stained with DHE evaluated using a flow cytometer. (**b**) Bar charts summarize the mean percentage ± SD of total intracellular ROS positive cells. (**c**) Graphical representation of the total protein carbonyl group content variation in the different tested groups. Representative data from three independent experiments are shown as mean ± SD (*n* = 3). An asterisk (*) indicates a comparison of all treated groups to untreated healthy cells. A hashtag (#) indicates a comparison of the PTP inhibitor treated groups to FFA-challenged cells. */#, *p* < 0.05, **/##, *p* < 0.01, ***/###, *p* < 0.001. HepG2_HE: HepG2 healthy untreated cells; HepG2_FFAs: HepG2 cells exposed to the palmitate/oleate combination; HepG2_MSI-1436: HepG2 cells pre-treated with 1 µM MSI-1436 inhibitor and exposed to the palmitate/oleate combination; HepG2_Comp 23: HepG2 cells pre-treated with 1 µM compound 23 inhibitor and exposed to the palmitate/oleate combination.

The accumulation of FFAs is known to mediate the excessive generation of ROS and trigger oxidative stress. In HepG2 cells treated (or not) with PTP inhibitors and intoxicated with the FFA combination, oxidative stress status was evaluated by recording the intracellular ROS using a Muse ^®^ Oxidative Stress Kit, as well as quantifying the total protein carbonylation levels via the DNPH derivatization method (Figure 5). Incubating HepG2 cells with the palmitate/oleate mixture significantly induced excessive intracellular ROS production, as evidenced by the high accumulation of FFA-treated HepG2 cells in the ROS positive cell population (Figure 5a). The lipid-overloaded cells exhibited almost a 1.75-fold higher percentage of ROS positive cells (Figure 5b) compared to the healthy cells control group (*p* < 0.001). As a consequence of intracellular ROS overproduction, the FFAs simultaneously induced the occurrence of carbonyl stress within HepG2 cells, as highlighted by the significant increase in protein carbonyl reactive species compared to the normal control group (*p* < 0.001), likely resulting from the oxidative stress-associated increased production of lipid hydroperoxides, which inevitably induce the oxidation of neighboring proteins and other molecules. HepG2 culturing with MSI-1436 and compound 23 prior to FFA addition attenuated oxidative stress and significantly lowered the percentage of cells overproducing ROS (Figure 5b). Moreover, the two PTP inhibitors prevented the carbonylation of HepG2 proteins, as shown by the nearly 1.5-fold reduction in total protein carbonyl fragments compared to FFA-stressed cells (*p* < 0.001).

### 3.6. PTP Inhibitors Attenuate FFA-Induced ER Stress in HepG2 Cells

**Figure 6:**
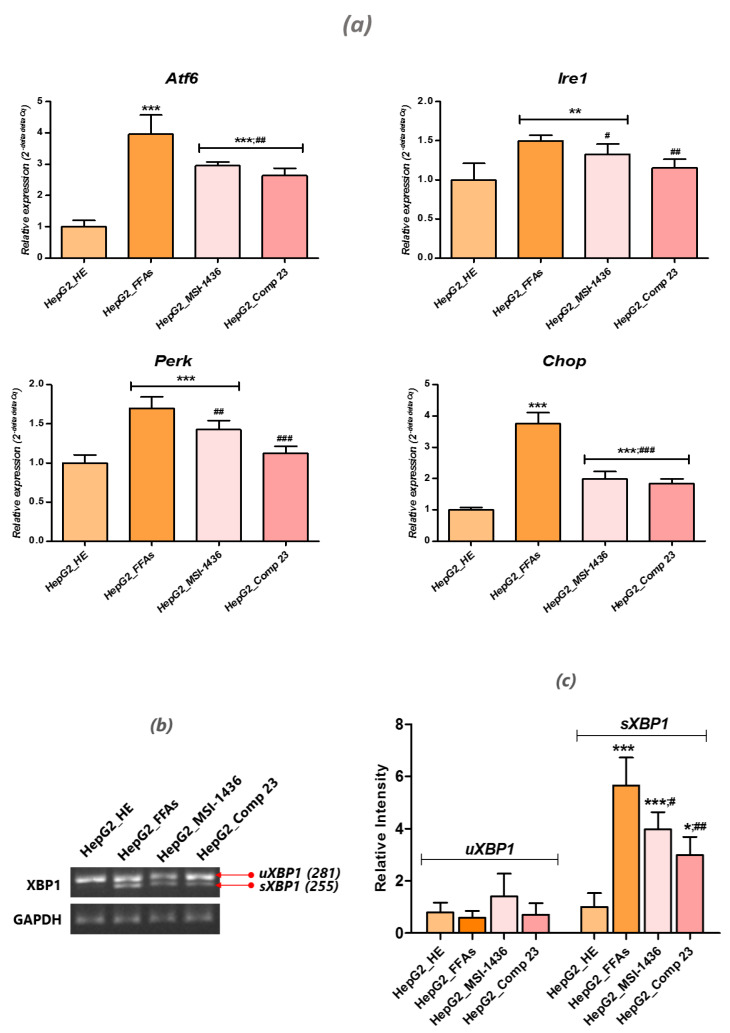
Effects of MSI-1436 and compound 23 on palmitate/oleate-induced ER-stress in HepG2 cells. (**a**) Bar charts summarize the mean relative expression ± SD of ER-stress related transcripts. (**b**) T-PCR product visualization of the uXBP1 and sXBP1 transcripts. (**c**) Histograms showing the relative intensity of x-box binding protein (uXBP1) and sXBP1 mRNA. Representative data from three independent experiments are shown as mean ± SD (*n* = 3). An asterisk (*) indicates a comparison of all treated groups to untreated healthy cells. A hashtag (#) indicates a comparison of PTP inhibitor treated groups to FFA-challenged cells. */# *p* < 0.05, **/## *p* < 0.01, ***/### *p* < 0.001. HepG2_HE: HepG2 healthy untreated cells; HepG2_FFAs: HepG2 cells exposed to the palmitate/oleate combination; HepG2_MSI-1436: HepG2 cells pre-treated with 1 µM MSI-1436 inhibitor and exposed to the palmitate/oleate combination; HepG2_Comp 23: HepG2 cells pre-treated with 1 µM compound 23 inhibitor and exposed to the palmitate/oleate combination.

Previous studies established that exogenous FFAs, such as palmitate, trigger ER stress by activating expression of the proapoptotic transcription factor *Chop* while modulating the *Akt* pathway [31]. In this regard, the present study aimed to investigate whether modulation of the PTP enzyme may impact the induction of ER stress within cultured HepG2 cells overloaded with the FFA mixture. A relative gene expression analysis of FFA-treated HepG2 cells revealed the overactivation of ER stress-related effectors (Figure 6a), as shown by the significant upregulation of *Atf6*, *Perk, Chop* and *Ire1* transcripts compared to normal untreated HepG2 cells (*p* < 0.001). Moreover, the quantitative estimation of spliced *Xbp1* (*sXbp1*) mRNA indicated higher relative intensity (*p* < 0.001) in the group exposed to the palmitate/oleate combination compared to healthy cells (Figure 6b). Cells pre-conditioned with MSI-1436 and compound 23 strongly prevented the induction of *Atf6*, *Perk*, *Chop and Ire1* gene expression (*p* < 0.001; *p* < 0.01) unlike the FFA-induced HepG2 cells (Figure 6a). ER stress mitigation was further substantiated through the marked reduction of *Xbp1* mRNA splicing (Figure 6b).

### 3.7. PTP Inhibitors Ameliorate the Free Fatty Acid Composition Changes in HepG2 Cells Challenged with the PA/OA Mixture

**Figure 7:**
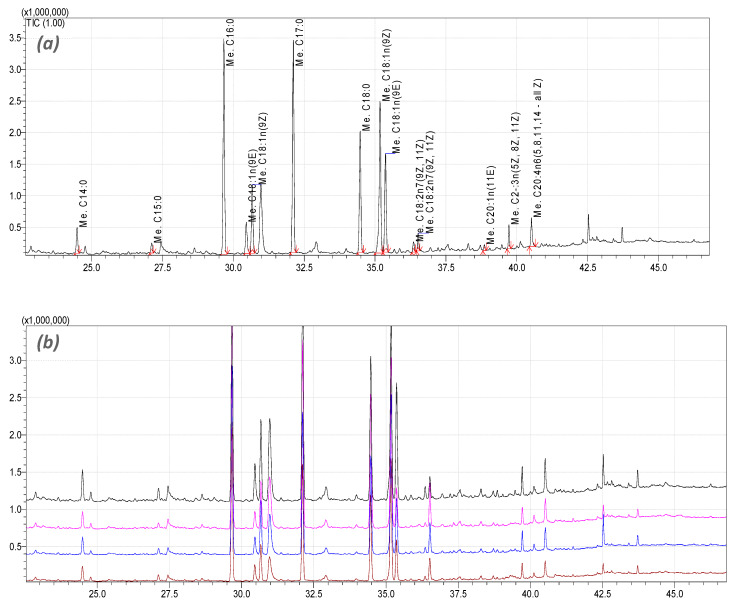
Gas chromatography–mass spectrometry (GC-MS) chromatograms for HepG2 lipidomic analysis. (**a**) Representative standard picks for each identified FFA. (**b**) The black signal indicates HepG2_HE: HepG2 healthy untreated cells; the pink signal indicates HepG2_FFAs: HepG2 cells exposed to the palmitate/oleate combination; the blue signal indicates HepG2_MSI-1436: HepG2 cells pre-treated with 1 µM MSI-1436 inhibitor and exposed to the palmitate/oleate combination; the brown signal indicates HepG2_Comp 23: HepG2 cells pre-treated with 1 µM compound 23 inhibitor and exposed to the palmitate/oleate combination.

A GC-MS-based lipidomic approach was undertaken to quantify the major lipid classes and the distribution of fatty acids within the FFA-fattened HepG2 cells treated with PTP inhibitors and healthy HepG2 cells (Figure 7). There was a trend toward a progressive decrease from the controls to FFA-treated HepG2 cells for n-6 and n-3 polyunsaturated fatty acids (PUFAs) within the hepatic FFAs. A significant increase in palmitic acid (C16:0, 21.37 ± 0.21 μg/10^7^ cells), stearic acid (C18:0, 11.66 ± 0.06 μg/10^7^ cells) and oleic acid (C18:1n, 5.10 ± 0.43 06 μg/10^7^ cells) in all treated groups was also observed (Table 2). As may be observed in Figure 7, the overall lipidomic profiles of all hepatocyte samples overlap. However, the specific amounts of FAAs differ across the samples. Table 2 illustrates that, as a consequence of the applied treatments, the FAA concentration increased clearly in five cases. The largest increase was observed for palmitic acid, stearic acid and oleic acid—the most abundant FAAs in healthy, untreated hepatocytes—and was followed by an increase of methyl octadecadienoate (C18:2n, 9Z,11E) and methyl palmitoleate (C16:1n, 9Z). For myristic acid (C14:0), methyl Pentadecanoate (C15:0), methyl elaidate (C18:1n, 9E) and methyl octadecadienoate (C18:2n, 9Z,11Z), a decrease in concentration was observed at various levels. Supplementation of the HepG2 cultures with PTP inhibitors (namely MSI-1436 and Compound 23) markedly reduced the intracellular levels of palmitoleate compared to the FFA-treated cells (*p* < 0.001). Moreover, increased levels of n-6 (arachidonic acid: 20:4n-6) and n-3 (eicosapentanoic acid: 20:5n-3, docosahexanoic acid: 22: 6n-3) were observed in the cultures supplemented with PTP inhibitors compared to the FAA-treated and untreated hepatocytes.

## 4. Discussion

Lipotoxicity results from the excessive accumulation and storage of FFAs in peripheral non-adipose tissues, such as the liver, pancreas, skeletal muscle and heart. A lipid overload subsequently leads to apoptotic cell death and a loss of functional tissue mass [32,33], which may further contribute to cellular dysfunction. It has been previously described that excess FFAs strongly impair insulin secretion and insulin gene expression [34] and promote mitochondrial dysfunction, ER stress, and ultimately, apoptosis [35]. More recently, consistent data have provided evidence that PTP1B and LMPTP are clearly involved in the pathophysiological mechanisms governing the development of lipotoxicity, cellular dysfunction and metabolic disorders affecting liver tissue. Moreover, a growing body of evidence suggests that decreasing PTP1B in various tissues, including the muscle, liver and brain, may lead to a multitude of beneficial effects, which indicates that PTPases inhibitors provide a promising tool for the management of different metabolic conditions, such as type 2 diabetes and cancer [12,36,37]. The purpose of the present investigation, therefore, was to determine whether PTP1B and / or LMPTP inhibition could prevent lipotoxicity in a HepG2 cell line challenged with a combination of palmitate and oleate free fatty acids.

Fatty acids are basically classified as saturated and unsaturated (monounsaturated and polyunsaturated). The biologic effects of fatty acids depend mainly on their structure. This particularly concerns the FAs commonly found in dietary food and serum, such as palmitic acid (PA), a saturated FA and oleic acid (OA), a monounsaturated FA, which are among the most abundant cellular fatty acids in mammals [38,39]. These FFAs are also believed to be major contributors to an increase in intrahepatic triglyceride and affect lipid homeostasis, which represents a pathophysiologic hallmark of NAFLD [40]. By using a lipidomic approach, the present investigation defined the amounts and types of metabolically derived lipids that accumulate within HepG2 cells after PTP inhibitors treatment and PA/OA supplementation. The GC-MS analysis of the HepG2 cell extract showed that the application of the PA/OA mixture induced significant changes in polyunsaturated fatty acid (PUFA) content compared to the healthy group of cells, which is a typical characteristic of NAFLD. HepG2 cells successfully developed a lipotoxic stat after PA/OA challenging, as evidenced by the significant increase in palmitic acid (C16:0), Palmitoleic acid (C18:1n) and Stearic acid (C18:0) in all treated groups. Supplementation of HepG2 cultures with PTP inhibitors (namely MSI-1436 and Compound 23) markedly reduced the intracellular levels of palmitoleate; high levels of the latter compound have been robustly associated with multiple metabolic risk factors, including lipotoxicity and NAFLD [41]. Palmitoleic acid derives from the conversion of saturated fatty acids, such as palmitoyl- and stearoyl-CoA, by Stearoyl-CoA desaturase (*Scd*), which is an endoplasmic reticulum membrane protein induced by different stimuli, such as glucose or saturated fatty acids [42]. Previous studies demonstrated that there is a significant correlation between *Scd* and PTP1B activities; the modulation of *Scd1* strongly decreases PTP1B protein levels, which was similarly observed in the muscle of *Scd1^−/−^*mice. Taken together with our obtained data, there is strong evidence that the inhibition of PTP1B can regulate the activity of *Scd1* and thus reduce the synthesis of toxic monounsaturated fatty acids (MUFAs) [43,44,45].

Previously, Puri et al. [46], reported that, during NAFLD onset, there are relevant modifications in the downstream n-6 (arachidonic acid: 20:4n-6) and n-3 (eicosapentanoic acid: 20:5n-3, docosahexanoic acid: 22: 6n-3) fatty acids in both NAFLD and NASH. Moreover, the authors suggested that the observed alterations in the product/precursor ratios for both the n-6 and n-3 pathways in FFA may correlate with the modified activities of Δ^6^-desaturase and Δ^5^-desaturase (which are known to catalyze the conversion of these precursor Essential fatty acids (EFAs) to downstream n-6 and n-3 fatty acids) over the course of NAFLD. On the other hand, the increased levels of conjugated linoleic acid (C18:2n7) and mead acid (20:3n-9) indicate that there was an essential fatty acid (EFA) deficiency in fattened HepG2 cells [46]. Arachidonic acid (20:4n-6) is known to be released from membrane phospholipids by phospholipase A2 and from phosphatidylinositol bisphosphate through DAG by phospholipase C to be rapidly converted by the cyclooxygenase into proinflammatory prostaglandins, thromboxanes and leukotrienes. Treatment of PA/OA-fattened HepG2 cells with the two PTP inhibitors induced an increase in total arachidonic acid levels. This phenomenon may be explained by a possible reduction of arachidonic acid utilization and a direct or indirect modulation of phospholipases and cyclooxygenase for the regulation of the inflammatory pathways that are involved in NAFLD [46,47]. In contrast, another study evidenced the dual effect of PTP1B over the course of NASH onset. PTP1B-KO mice challenged with a methionine/choline-deficient diet (MCD) for hepatic focal inflammation induction exhibited increased steatosis compared to the PTP1BWT controls. Deficiency in PTP1B resulted in a higher expression of the M1 responsive genes in macrophages loaded with palmitate. Likewise, the M1 proinflammatory cytokine transcript levels were markedly elevated in PTP1B-KO livers from MCD-fed mice.

However, when MCD was switched to a chow diet (CHD), the PTP1BKO mice rapidly reverted to NASH compared to the PTP1BWT mice in parallel with the normalization of serum triglycerides levels. Additionally, a drop in the cytotoxic natural killer T subpopulation was monitored in PTP1BKO livers during recovery concomitantly with M2 macrophage marker up-regulation [48]. These findings suggest that, as a possible dual effect, under PTP1B inhibitory conditions, excessive arachidonic acid release may be triggered while reversing HepG2 cell metabolic status, thereby suggesting that a homeostatic balance in PTP1B expression and activity is necessary for proper cellular functioning. This hypothesis should, however, be confirmed or rejected through further investigations.

Many previous studies clearly established that the overaccumulation of PA and OA FFAs inevitably triggers HepG2 cell death and apoptosis in a dose-dependent manner [49,50,51]. Furthermore, it has been recently postulated that the expression level of PTB1B may exert a pivotal role in maintaining the balance between survival and death in hepatocytes [52,53,54]. Many in vitro experiments also demonstrated that some factors, such as PA and OA, may up-regulate PTP1B expression and thus lead to the appearance of IR and apoptosis in these cells [55,56,57]. In this study, treatment of HepG2 cells with the PA/OA mixture resulted in a sharp increase of the total apoptotic population, which was accompanied by a significant upregulation in the mRNA expression levels of the pro-apoptotic factors *p53*, *p21*, *Bax*, *caspase-3* and *caspase-9*, while *Bcl-2* expression appeared to be highly decreased compared to the untreated cells. Moreover, FFA application significantly induced the activation of different caspase complexes that are widely involved in apoptosis cascades. The obtained results demonstrate that pretreatment with MIS-1436 and compound 23 can prevent PA/OA-induced hepatocyte apoptosis and significantly restore the down-regulated expression of *Bcl-2* while decreasing the upregulated expression of *p53*, *p21*, *Bax*, *caspase-3* and *caspase-9* compared to non-treated cultures supplemented with FFAs. These data suggest that the pretreatment of HepG2 cells with the two PTP inhibitors prior to PA/OA elicitation may confer a protective effect against Lipotoxicity-induced hepatocytes apoptosis. Our findings appear to be consistent with previous investigations that demonstrated that the absence of PTP1B markedly protects against FA-induced massive liver apoptosis and fulminant hepatic failure in vivo. Moreover, the injection of adenovirus expressing PTP1B into PTP1B-KO mice obviously induced a higher elevation of circulating transaminase liver enzymes than adenovirus alone, indicating that elevated levels of PTP1B may potentiate adenovirus induced hepatic damage and liver cell death. This highlights that protection from apoptosis in the liver occurs at the level of the initiator caspase-8 through the cleavage of caspase-8, as well as downstream caspases-3 and -9, which seemed to be abrogated in PTP1B-null mice [13,54,58]. PTP1B deficiency in a model of PTP1B-KO mice strongly improved liver regeneration after partial hepatectomy and protected hepatocytes against cell death, indicating a higher proliferative/survival capacity of liver cells in the absence of PTP1B. The proposed molecular mechanism responsible for this effect likely involves the restoration of tyrosine kinase receptors, such as the EGF receptor, HGF receptor (Met) or insulin-like growth factor-1 receptor (IGF1R), which are known to be closely implicated in the control of hepatocyte proliferation and survival and modulated through PTP1B-induced dephosphorylation [48]. Obtained results demonstrated also that LMPTP inhibition using its selective modulator (Compound 23), can successfully prevent and reduce lipids-induced apoptosis in liver. Similarly, Alicka et al. [59], reported on the beneficial effect of LMPTP inhibition on equine hepatic progenitor-like cells (HPCs) survival in the course of PA-induced lipoapoptosis, evidenced by the observed changes in calcein-acetoxymethyl (AM) / propidium iodine (PI) fluorescence intensity. Taken together, these data indicate that LMPTP is also implicated in regulating cell survival during cellular metabolic disruption.

A number of in vitro studies indicated that palmitate alone or in combination with other FFAs, such as oleate, induces oxidative stress and triggers significant mitochondrial DNA damage that correlates to concomitant mitochondrial dysfunction, apoptosis and the inhibition of insulin signaling [50,60,61,62]. The inhibition of PTPs using both MSI-1436 and compound 23 successfully maintained the mitochondrial dynamics that were strongly impaired after PA/OA challenging through an increase in mitochondrial membrane potential, the up-regulation of mitochondrial fusion genes (*Pink1* and *Mnf*), and the suppression of genes that are involved in mitochondrial fission, namely *Mff* and *Fis1*. Nonetheless, it should be noted that the present investigation has certain limitations due to the lack of mitochondrial protein profiling, as well as the study of isolated mitochondrial fractions, which would provide further data on the precise mechanisms of action involves in the process.

Previous findings showed that the overexpression of PTP1B over the course of FFA accumulation strongly downregulated PGC-1, while PTP1B knockdown prevented this reduction. The protective effect of PTP1B inhibition on the decline in ATP levels has also been reported, suggesting that PTP1B modulation positively affects mitochondrial function [63,64].

On the other hand, it was shown that *Sirt1*, which is an important upstream regulator of PGC-1, can be impaired following FFA challenging and that the resulting overexpression of PTP1B lies at the origin of decreased *Sirt1* protein levels, while its inhibition promoted the expression of *Sirt1*. These observations highlight the fact that PTP1B acts as an upstream regulator of *Sirt1* and that its inhibition provides a valuable strategy for the restoration and maintenance of mitochondrial homeostasis [65,66]. For its part, LMPTP inhibition has been previously demonstrated to promote mRNA levels of *Fis1* and *Prkn* transcripts, without affecting expression rate of *Mfn1* and *Pink1* in a model of HPCs challenged with palmitate, highlighting a potential regulatory role of LMPTP on mitochondrial homeostasis [59]. Oxidative stress clearly plays a critical role in the development of liver fibrosis. Excess FFAs critically trigger the overgeneration of reactive oxygen species (ROS), subsequently inducing lipotoxicity, which is ultimately associated with the appearance of ER stress, calcium dysregulation, mitochondrial dysfunction and cell death [67]. In the present study, HepG2 cells challenged with the PA/OA combination were markedly prone to intracellular ROS overproduction, as well as high levels of total protein carbonyl moieties, as a consequence of induced lipotoxicity. The pre-conditioning of FFA-induced cultures with PTP inhibitors significantly lowered the levels of ROS-positive cells, as well as the amount of intracellular carbonyl groups. Similarly, both inhibitors were able to modulate ER stress induced by the intracellular overaccumulation of FFAs, as evidenced by the down-regulation in the expression of ER stress-related transcripts and the reduction in spliced *xbp1*. ER-stress response and PTP1B expression are known to be closely interlinked; it has been shown that the downregulation of PTP1B expression in the liver can relieve overactivation of the ER-stress response associated with HFD feeding, obesity and insulin resistance [36]. PTP1B importantly contributes to the ER-stress response, as liver-specific deficiency in mice or the siRNA knockdown in hepatic cells modulated the full activation of all three ER-stress pathways, *Ire1-α/Xbp-1*, *Perk/eIF2α* and *Atf6* [12,68]. Collectively, these studies suggest that there may be a positive feedback loop between PTP1B expression and full activation of the ER-stress response and that inhibition of PTP1B facilitates the alleviation of ER stress in HepG2 cells [12]. Moreover, strong correlation has been demonstrated in the course of the present study between LMPTP activity and ER-stress, implying that this enzyme may also be involved in additional cellular metabolic pathways. These observations are in agreement with previous established data, that revealed a positive ER-stress regulation upon LMPTP inhibition, as evidenced by the diminished expression of *Chop*, *Atf6* and *Hspa5* transcripts, as well as decreased splicing of *Xbp1* and increased *Ire1α* endonuclease activity, in PA-induced lipotoxicity in HPCs cells [59]. The observed regulatory effect on UPR-related genes following PTP1B and LMPTP inhibition should however be extended and analyzed at the protein level.

## 5. Conclusions

Lipotoxicity results from the overaccumulation of FAAs within liver tissue and represents one of the most common hallmarks of NAFLD. In this study, we analyzed the effect of two PTP inhibitors in an experimental model of lipotoxicity induced by a mixture of PA and OA. Our data demonstrate that the altered apoptosis caused by PTP1B and LMPTP elicitation in HepG2 cells was successfully abrogated following cell pretreatment with the two inhibitors. Moreover, the inhibition of both PTPases in the hepatocytes resulted in decreased intracellular ROS production, the restoration of mitochondrial dynamics and the mitigation of induced ER stress. These findings further support that the inhibition of hepatic PTP1B and LMPTP contributes to the improvement of liver metabolism and may provide new insights into the development of valuable therapeutic strategies to prevent the lipotoxic states that occur in NAFLD. Nevertheless, the implementation of more in-depth experiments dealing with, for example, proteomic analyses would allow us to refine the exact molecular mechanisms of action.

## Figures and Tables

**Table 1:** Sequences of primers (Sigma Aldrich, Poznań, Poland) used in qPCR.

Gene	Primer	Sequence 5′–3′	Amplicon Length (bp)	Accession No.
*Mnf1*	F: R:	GTTGCCGGGTGATAGTTGGA TGCCACCTTCATGTGTCTCC	146	NM_033540.3
*Fis1*	F: R:	TGGTGCGGAGCAAGTACAAT TGCCCACGAGTCCATCTTTC	132	NM_016068.3
*Pink1*	F: R:	GCTTGGGACCTCTCTTGGAT CGAAGCCATCTTGAACACAA	142	NM_032409.3
*Mff*	F: R:	TCTCAGCCAACCACCTCTGA TGAGAGCCACTTTTGTCCCC	109	NM_001277061.2
*Atf6*	F: R:	ACCTCCTTGTCAGCCCCTAA CACTCCCTGAGTTCCTGCTG	150	NM_007348.4
*Perk*	F: R:	TGCTCCCACCTCAGCGAC TTTCAGGATCCAAGGCAGCA	124	NM_004836.6
*Chop*	F: R:	TAAAGATGAGCGGGTGGCAG GGATAATGGGGAGTGGCTGG	103	NM_001195053.1
*Ire1*	F: R:	CGGCCTCGGGATTTTTGGA AGAAAGGCAGGCTCTTCCAC	110	NM_001433.5
*Xbp1*	F: R:	TTACGCGAGAAAACTCATGGCC GGGTCCAAGTTGAACAGAATGC	281 (unspliced) 255 (spliced)	XM_014742035.2
*p53*	F: R:	AGATAGCGATGGTCTGGC TTGGGCAGTGCTCGCTTAGT	381	NM_001126118.1
*Bax*	F: R:	ACCAAGAAGCTGAGCGAGTGTC ACAAAGATGGTCACGGTCTGCC	356	XM_011527191.1
*Bcl-2*	F: R:	ATCGCCCTGTGGATGACTGAG CAGCCAGGAGAAATCAAACAGAGG	129	NM_000633.2
*p21*	F: R:	AGAAGAGGCTGGTGGCTATTT CCCGCCATTAGCGCATCAC	169	NM_001220777.1
*Casp3*	F: R:	CTCTGGTTTTCGGTGGGTGT CTTCCATGTATGATCTTTGGTTCC	136	NM_004346.4
*Casp9*	F: R:	CAGGCCCCATATGATCGAGG CTGGCCTGTGTCCTCTAAGC	142	NM_032996.3
*GAPDH*	F: R:	GTCAGTGGTGGACCTGACCT CACCACCCTGTTGCTGTAGC	256	NM_001289746.1

Mfn1: Mitofusin 1; Fis1: Mitochondrial fission 1 molecule; Pink1: PTEN-induced putative kinase 1; Mff: Mitochondrial fission factor; Atf6: Activating transcription factor 6; Perk: PRKR-like endoplasmic reticulum kinase; Chop: C/EBP homologous protein; Ire1: Inositol-requiring enzyme; Xbp1: X-box binding protein 1; P53: tumor suppressor p53; Bcl-2: B-cell lymphoma 2; Bax: BCl-2 associated X protein; p21: Cyclin-dependent kinase inhibitor 1; Casp3: Caspase 3; Casp9: Caspase 9; GADPH: Glyceraldehyde-3-phosphate dehydrogenase.

**Table 2:** Fatty Acid Composition of the FFA Lipid Class in HepG2 treated and untreated cells.

*FFAS*	*HEPG2_HE* (µG)	*HEPG2_FFAS* (µG)	*HEPG2_MSI-1436* (µG)	*HEPG2_COMP 23* (µG)
*TETRADECANOIC ACID, METHYL ESTER; ME. C14:0*	3.00 ± 0.05	2.16 ± 0.01 ***	2.85 ± 0.02 **^,###^	2.96 ± 0.03 ^###^
*PENTADECANOIC ACID, METHYL ESTER; ME. C15:0*	1.05 ± 0.00	0.60 ± 0.01 ***	0.83 ± 0.01 ***^,###^	1.07 ± 0.02 ^###^
*HEXADECANOIC ACID, METHYL ESTER; ME. C16:0*	21.37 ± 0.21	26.86 ± 0.24 ***	27.86 ± 0.34 ***^,#^	28.50 ± 0.26 ***^,##^
*9-HEXADECENOIC ACID, METHYL ESTER, (E)-; ME. C18:1N(9Z)*	1.08 ± 0.10	0.64 ± 0.05 ***	0.95 ± 0.06 ^##^	1.14 ± 0.03 ^###^
*9-HEXADECENOIC ACID, METHYL ESTER, (Z)-; ME. C18:1N(9Z)*	2.28 ± 0.20	1.80 ± 0.13 *	2.77 ± 0.16 *^,###^	2.55 ± 0.03 ^##^
*OCTADECANOIC ACID, METHYL ESTER; ME. C18:0*	11.66 ± 0.06	15.38 ± 0.07 ***	14.23 ± 0.06 ***^,###^	15.29 ± 0.06 ***
*9-OCTADECENOIC ACID (Z)-, METHYL ESTER; ME. C18:1N(9Z)*	5.10 ± 0.43	6.23 ± 0.45	7.92 ± 0.53 **^,#^	8.27 ± 0.03 ***^,##^
*9-OCTADECENOIC ACID, METHYL ESTER, (9E); ME. C18:1N(9E)*	2.82 ± 0.06	1.28 ± 0.02 ***	2.40 ± 0.02 ***^,###^	2.09 ± 0.02 ***^,###^
*9,11-OCTADECADIENOIC ACID, METHYL ESTER, (9Z,11Z); ME. C18:2N7(9Z, 11Z)*	0.35 ± 0.01	0.16 ± 0.01	0.29 ± 0.02	0.18 ± 0.12
*9,11-OCTADECADIENOIC ACID, METHYL ESTER* *, (9Z,11Z); ME. C18:2N7(9Z, 11Z)*	0.65 ± 0.01	1.75 ± 0.02 ***	1.65 ± 0.03 ***^,##^	1.51 ± 0.01 ***^,###^
*11-EICOSENOIC ACID, METHYL ESTER. (11E); ME. C20:1N(11E)*	0.12 ± 0.02	0.00 ± 0.00 **	0.04 ± 0.05	0.00 ± 0.00 **
*5,8,11-EICOSATRIENOIC ACID, METHYL ESTER, (5Z,8Z,11Z); ME. C2:3N(5Z,8Z,11Z)*	0.48 ± 0.02	0.44 ± 0.02	0.64 ± 0.04 **^,###^	0.61 ± 0.02 **^,##^
*5,8,11,14-EICOSATETRAENOIC ACID, METHYL ESTER, (ALL-Z) (ME. C20:4N6(5,8,11,14—ALLZ)*	0.67 ± 0.03	0.69 ± 0.02	0.91 ± 0.05 ***^,###^	0.74 ± 0.01 *^,#^

Representative data from three independent experiments are shown as mean ± SD (*n* = 3). An asterisk (*) indicates a comparison of all treated groups to untreated healthy cells. A hashtag (#) indicates a comparison of the PTP inhibitor treated groups to FFA-challenged cells. */#, *p* < 0.05, **/##, *p* < 0.01, ***/###, *p* < 0.001. HepG2_HE: HepG2 healthy untreated cells; HepG2_FFAs: HepG2 cells exposed to the palmitate/oleate combination; HepG2_MSI-1436: HepG2 cells pre-treated with 1 µM MSI-1436 inhibitor and exposed to the palmitate/oleate combination; HepG2_Comp 23: HepG2 cells pre-treated with 1 µM compound 23 inhibitor and exposed to the palmitate/oleate combination.

## Abbreviations

7-AAD	cid Phosphatase 1
AM	Acetoxymethyl
DAG	Diacylglycerol
DNPH	2,4-Dinitrophenyl Hydrazine
EFAs	Essential Fatty Acids
FAMEs	Fatty Acid Methyl Esters
FFAs	Free Fatty Acids
GC-MS	Gas chromatography–mass spectrometry
HBSS	Hanks’ Balanced Salt solution
HCC	Hepatocellular Carcinoma
HepG2	Human hepatocarcinoma
HER	Human Epidermal Growth Factor Receptor
HFD	High-Fat diet
HPCs	Equine Hepatic Progenitor-like Cells
HTN	Systemic Hypertension
INRS	Insulin Receptor
JAK2	Janus Kinase 2
KO	Knockout
LMPTP	Low Molecular Weight Protein Tyrosine Phosphatase
MetS	Metabolic Syndrome
MSI-1436	Trodusquemine
MUFAs	Monounsaturated Fatty Acids
NAFLD	Non-alcoholic Fatty Liver Disease
NASH	Nonalcoholic Steatohepatitis
OA	oleate
PA	palmitate
PGC-1	Peroxisome Proliferator-activated Receptor γ Coactivator 1
PI	Propidium Iodide
Prkn	Parkin RBR E3 Ubiquitin Protein Ligase
PTP1B	Protein-tyrosine phosphatase 1B
PTPN1	Protein Tyrosine Phosphatase Non-receptor type 1
PTPs	Protein Tyrosine Phosphatases
PUFA	Polyunsaturated Fatty Acids
ROS	Reactive Oxygen Species
RT-PCR	Reverse transcription Polymerase Chain Reaction
SCD	Stearoyl-CoA Desaturase
T2DM	Type 2 Diabetes
UPR	Unfolded Protein Response
